# The Late John Newcom, M. D.

**Published:** 1857-03

**Authors:** S. S. Satchwell

**Affiliations:** New Hanover county, North Carolina


ATLANTA
Medical and Surgical Journal.

VOL. II.] MARCH, 1857. [NO. 7

### ORIGINAL COMMUNICATIONS.

### ARTICLE I.

*The late* JOHN NORCOM, M. D. By S. S. Satchwell, M. D., of New Hanover county, North Carolina.

DIED, in the town of Edenton, North Carolina, on the 22d of January, 1856, of pulmonary apoplexy, JOHN NORCOM, M. D., in the fifty-fifth year of his age.

He was a man of mark in the profession and in the community. Frank, educated, and high-minded as a man, he was skillful, high-toned, and accomplished as a physician. I knew him long and well—I was his office pupil. Then, and always afterwards, he honored me with his confidence and friendship. I am unwilling that a physician of his character, and a man of his undisputed worth, should pass away unnoticed.

It is due to the profession, that the character and services of our leading medical men should be made public.

The life of the physician is one of gentleness and a devotion to ministrations for the relief of suffering humanity, rather than of show and selfish aims. They know but little of the properly educated physician, who charge that his great aim is money. He must eat and drink, and love an independent subsistence like other men, it is true; but the cares and anxieties and labors of such a man are based on higher motives, nobler impulses than the love of mammon.

In the hour of danger, he is greeted with warm and grateful welcome; in seasons of pestilence, he is hailed as the benefactor of his race. But when these trying times are over, how soon are his services forgotten; and when he dies, how soon does his name become cold in the hearts and upon the lips of men. Look around you in society, and who are the men that receive the honors and emoluments of life? Noisy political demagogues, factitious statesmen, visionary reformers, cunning speculators, and other pretenders in the various walks of life. These are the men who are crowned with popular applause, and with what the world calls success, while the real benefactors of our race, the men of science and worth, and honor, scorning to degrade themselves by using unworthy means of advancement, are elbowed aside by these pressing throngs of unscrupulous aspirants for power and wealth. In such a contest, and in such an age of restlessness, and shallow pretensions, the physician of genuine worth and merit, is apt to live unrewarded and to die unnoticed.

But it is not our purpose to dwell on these things, nor to descant on the merits of the medical profession farther than to remark, that no profession is so laborious and self-sacrificing as ours, and none so poorly rewarded in purse and public reputation.

Dr. Norcom was born and raised in Edenton, N. C. He was educated under the watchful eye of his father, Dr. James Norcom, an excellent scholar, and one of the most distinguished physicians of his day. John Norcom graduated with honor in the collegiate department of the University of Pennsylvania. He studied medicine under his father, and graduated in the medical department of the University of Baltimore. He then practiced with his father a year or two, deriving great advantage from his experience. He then removed to Washington county, and practiced with success. He gained here, rapidly upon public favor; but he desired a better field for his talents; and in a year or two more removed to the town of Washington, in Beaufort county. There he soon took the lead in his profession. His skill and unwavering attachment to his profession, which ever characterized him, secured him a large practice, and many friends. But his young ambition urged him to seek a still better field of exertion. He removed to Baltimore, under favorable circumstances, and commenced practice. He did well. His social and professional walks in this city, were in the front ranks of the profession. But a severe and contagious disease brought affliction in his family. His wife became dissatisfied with Baltimore. His relatives and friends in North Carolina were strong and incessant in their petitions to him to return. He yielded. He gave up all his flattering prospects in Baltimore and returned to the town of Washington. He soon regained the large and profitable practice he had previously left, and re-instated himself as the leading physician in that whole section of country. This position he continued to maintain as long as he lived. He was well worthy of the distinction.

His fondness for his profession was proverbial. It caused him to store his mind, well disciplined by early training, with a vast amount of professional learning. The doctrines of the older authors, as well as the views of modern writers and cotemporary practitioners were familiar to him. He kept himself well posted up in progressive medicine. With a mind thus learned and devoted, he brought to the bedside, a power of analysis, an accuracy of examination, and a judgment in the application of remedies, rarely surpassed. It was this fine discrimination, this power of analysis, and of applying remedies accordingly, that gave to him, as to every successful practitioner, such fine success in the treatment of disease. Hence it was, that his counsels were so often invoked in difficult and complicated cases far and near. It was on such occasions, so well calculated to try the metal of the physician, that Dr. Norcom was always ready. He was, indeed, a blessing to the community. Often, when superficial medical minds were torturing instead of relieving the patient by prescribing for *effects* and ambiguous symptoms, would he apply his critical mind to the investigation of the case, discover the *causes*, often hidden, and obscure, and by an appropriate corresponding treatment, cure the case, and bear away the palm of victory. But he would never use it to the injury of the attending physician; rather would he seek to bolster him up, unless his conduct was intolerably outrageous. Often has it happened, that patients, afflicted with some severe and lingering chronic disease, have gone the rounds among regular and irregular practitioners, sometimes remaining for months under the treatment of some celebrated physician at the North, and at last, without relief and in the agony of despair, have applied to Dr. Norcom, and were cured by him. But Dr. Norcom lived in plain old North Carolina—too artless and honest to proclaim her renown in the popular bugle blasts of the times—and he never stood at the corners of streets to publish his own deeds; and hence, such triumphs of skill are unknown abroad.

When not engaged in active practice, he was at his office poring over his books—studying his cases, or in the bosom of his family instructing his children. He had no time for light company, or the frivolous pleasures of the day. That time, which so many give to such frivolities, was appropriated by him, to more intellectual enjoyments, and to nobler purposes. His office was never sought by idlers, politicians, or demagogues: Dr. Norcom was not the man to give countenance to such characters. His friends were select, and no man enjoyed the company of his friends, at proper seasons, with a better relish, than he did. In refined and cultivated social circles, he was at home, and the delight of the company.

I have said, that in the hour of danger, hope and confidence settled upon him. At such times, even his bitter, personal enemies invoked his assistance, and he cheerfully extended it. In difficult cases, he was sent for far and near, to obtain his services in consultation. His practice was extensive, and he applied himself to it with much zeal and perseverance.

He had an elevated sense of the duties and responsibilities of his profession; he did all in his power, to add to its elevation and dignity; he disdained everything that was low and mean, both in and out of the profession; and he rebuked imposture and impertinence wherever he found either. He scorned those little tricks and artifices, so often used by narrow minds and mean men, to obtain practice. He often incurred the hatred of such men as a matter of course; but, he pursued the even tenor of his way, and made no compromises with demagogueism, dishonor, or dishonesty. His was the open, free, generous, independent spirit. Whatever his own judgment and conscience conceived to be right, he did and said; never enquiring what effect it would have upon his own popularity—he relied on his own worth and merit for success.

To his own brethren in the profession, he was kind, courteous and honorable. It was only in cases where the common feelings of professional honor and brotherhood were grossly infringed upon, by some empirical pretender, or diplomæd quack, that the vials of his wrath were stirred, in a professional sense. To the younger members of the profession, especially just starting in life, he was kind and encouraging; ever ready to aid them with advice and sympathy, and by putting into their hands profitable practice. He did not believe that gray hairs constituted wisdom, or in the anti-republican doctrine of hereditary transmission of practice. But, whenever any young medical man of honor, talent and merit, settled in the same town, or near him, however humble his origin, or oppressed by poverty, Dr. Norcom sought him out and did all he could to advance him. That deference which others have for the aristocracy of wealth or blood, was never felt nor shown by him; but the aristocracy of mind and merit secured his homage and esteem. He was emphatically a man of *principle*. For this, he was ready, with Roman firmness, to battle from the first to the last, and always; he would make no concessions to what is called policy, and no compromises with injustice or meanness, come from what quarter they might. He could look with no favor upon wealth or distinction obtained by intrigue or foul means.

In politics, he was never a partisan, but a patriot, warmly devoted to the interests of his native State and country. The dirty work of politicians; the degrading character of party tactics; the dishonorable means of all parties to obtain success, were so disgusting and revolting to his elevated feelings and high character, that he would never be a working member of any party, but sometimes voted with one party, and then again with another. He never believed that subserviency to party yoke was an element of patriotism. With his talents, he could have wielded vast political influence, if he had consented to sacrifice his independence and become a pliant party man.

Whatever affected the community in which he lived, he was always interested in, and manifested it by his advocacy of all those measures of moral and sanitary reform, that were for good. The cause of education secured his active exertions; and his benevolence was aroused by whatever was proposed for the moral, educational and religious interests of mankind.

Dr. Norcom committed the fault, common to the profession in North Carolina, of writing but little for the medical press; and, yet, he was one of the best writers in our State. Much has been lost to the profession by this omission on his part, to record his views, derived from a long and discriminating observation and experience, in the practice of medicine. He conversed with much fluency and ease, and was a lucid, logical and forcible speaker. His powers of writing and speaking, added to his acquirements and professional enthusiasm, would have enabled him to adorn a Professor's Chair, in any of the Medical Colleges. More than one application of this sort was declined by him. He preferred the private practice of his profession in the old North State.

His character, as may readily be supposed, made him strong friends and bitter enemies. The former clung to him through evil and through good report. The latter, now that he is gone, will not deny to him, good intentions, incorruptible integrity, superior talents, lofty purposes and elevated principles. In his friendships, he was sincere, reliable and disinterested; and when he made a friend, he adhered to him with idolatrous devotion. In this respect, and in his relations with his family—his love for his father, mother, brothers and sisters—his affection for his wife and children, I have never known a man to possess more fidelity, more attachment, more purity and ardor of love and affection. He evinced this by his deep interest in all that concerned them; his gentle, tender nature towards them, and his ever active efforts to advance their welfare and happiness.

His death was sudden, but so tranquil and resigned, as to give a reasonable hope to his friends, that he had made his peace with his God. He was sick but an hour or two. When suddenly and violently seized, his mother rushed to his bedside, and enquired what she should do for him. "Nothing," he answered, "Nothing. I am in the hands of a mericiful God, whom I have feared and loved—He has called me, and I must go." And in a short time, before the services of a physician could be obtained, he expired. Thus he died, and may it have been so ordered that he had quenched the darts of the wicked one, on the shield of Faith.

